# A Potential Role of Bone Morphogenetic Protein 7 in Shell Formation and Growth in the Razor Clam *Sinonovacula constricta*

**DOI:** 10.3389/fphys.2020.01059

**Published:** 2020-08-28

**Authors:** Jiaxi Zhao, Baoyue Cui, Hanhan Yao, Zhihua Lin, Yinghui Dong

**Affiliations:** Zhejiang Key Laboratory of Aquatic Germplasm Resources, College of Biological & Environmental Sciences, Zhejiang Wanli University, Ningbo, China

**Keywords:** *Sinonovacula constricta*, *BMP*7, SNP, growth traits, association, RNAi

## Abstract

Bone morphogenetic proteins (BMPs) not only play essential roles in bone development but also are involved in embryonic growth, organogenesis cell proliferation and differentiation. However, the previous studies on the functions of shellfish BMPs genes are still very limited. To better understand its molecular structure and biological function, BMP7 of the razor clam *Sinonovacula constricta* (*Sc-BMP*7) was cloned and characterized in this study. The full length of *Sc-BMP7* is 2252 bp, including an open reading frame (ORF) of 1257 bp encoding 418 amino acids. The protein sequence included a signal peptide (1–32 aa), a prodomain (38–270 aa) and a TGF-β domain (317–418 aa). The quantitative expression of eleven adult tissues showed that *Sc-BMP*7 was significantly higher expressed in the gill, foot, and mantle (*P* < 0.05), but lower in hemocytes and hepatopancreas. In the early development stages, low expression was detected in the stages of unfertilized mature eggs, fertilized eggs, 4-cell embryos, blastula, gastrulae, whereas it increased after the stage of trochophore and demonstrated the highest expression in umbo larvae (*P* < 0.01). In shell repair experiment, *Sc-BMP*7 showed increasing expression level after 12 h. The higher expression of *Sc-BMP*7 was detected while Ca^2+^ concentration was reduced in seawater. After inhibiting *Sc-BMP*7 expression using RNA interference (RNAi) technology, expression of *Sc-BMP*7 mRNA and protein were significantly down-regulated (*P* < 0.05) in the central zone of mantle (nacre formation related tissue) and the pallial zone of mantle (prismatic layer formation related tissue). Association analysis identified two shared SNPs in exon of *Sc-BMP*7 gene from 246 individuals of two groups. These results indicated that *BMP*7 might be involved in shell formation and growth. These results would contribute to clarify the role of *Sc-BMP*7 in the regulation of growth and shell formation, and provide growth-related markers for molecular marker assisted breeding of this species.

## Introduction

Transforming growth factor-β (TGF-β) proteins comprise a family of structurally related cytokines that occur widely among various vertebrates and invertebrates. TGF-β is known to be involved in various biological processes, including bone and organ formation, cell proliferation, differentiation, apoptosis, and so on (Feng and Derynck, 2005; Massague, 2008; Ramesh et al., 2009). The TGF-β superfamily is divided into two main categories: TGF-β/activin/nodal and bone morphogenetic protein (BMP)/differentiation factor (GDF)/Müllerian inhibiting substance (MIS) (Shi and Massagué, 2003), based on sequence identity and activation of downstream pathways. All TGF-β superfamily members split from a precursor at a specific site to release a mature polypeptide, and their biological activity relies on the formation of dimers by two identical or different subunits (Kingsley, 1994). Among these proteins, BMPs constitute the largest subgroup of the TGF-β superfamily (Bragdon et al., 2011). BMPs are extensively expressed during mammalian development, with a wide range of biological activities, including development, proliferation, and extracellular matrix synthesis (Salazar et al., 2016). BMPs can distinguish and bind to serine/threonine kinase receptors, with subsequent signaling mediated by both Smad-dependent and -independent pathways (Wordinger and Clark, 2007; Sánchez-Duffhues et al., 2015).

In mollusks, the whole-body growth was determined by growth of soft parts and shell, the latter involving crystal growth regulated by the secretion of stromatin. Both shell and bone are products of biomineralization. Proteins account for <5% of the biomineralized shell but are primarily responsible for controlling the CaCO_3_ polymorph and texture (Marin and Luquet, 2004; Liu and Li, 2013). Most studies of BMPs in vertebrates have focused on *BMP*2, *BMP*4, and *BMP*7 (Salazar et al., 2016). However, other studies have shown that BMPs in mollusks also have important functions (Dong, 2012; Feng et al., 2013; Lin, 2014; Liu et al., 2014; Yan et al., 2014; Qian, 2015; Zhou, 2016; Fan et al., 2018), similar to higher animals.

BMP7 possesses the classical TGF-β domains, including a large pro-domain that facilitates protein folding and a mature signaling peptide (Xu et al., 2018), and plays important roles during skeletogenesis and postnatal bone homeostasis (Cook and Rueger, 1996). Mammalian BMP7 has been shown to induce the differentiation of primitive osteoblast progenitor cells and accelerate the healing of fractures (Schmal et al., 2012). Recombinant human BMP7 protein has been used in the clinic to promote bone regeneration (Asahina et al., 1996; Han et al., 2008; Hurtig et al., 2009). BMP7 has also been shown to play significant roles in the development of various types of tissues, such as kidney and brown adipose tissue. In mice, BMP7 was involved in sertoli cell proliferation during early postnatal development, and *BMP*7 gene knockout mice caused infertility (Puglisi et al., 2004; Monsivais et al., 2017). BMP7 has also been shown to participate in embryogenesis, tissue growth, and neurogenesis (Bragdon et al., 2011; Kowtharapu et al., 2018). The *BMP*7 gene was also identified as a candidate gene related to growth in cattle and chickens, and growth-associated single nucleotide polymorphisms (SNPs) have been identified (Chen et al., 2013; Huang et al., 2013; Wang et al., 2018). So far, *BMP*7 genes have been reported in some species of bivalves, including *Tegillarca granosa* (Dong, 2012), *Pinctada martensii* (Yan et al., 2014; Fan et al., 2018), and *Hyriopsis cumingii* (Lin, 2014). These studies also found the highest expression levels of *BMP*7 in the mantle, suggesting that it was related to shell formation.

The razor clam *Sinonovacula constricta* is an economically important maricultured bivalve species with over 800,000 metric tons of annual production in China (Xu and Zhang, 2008). However, despite recent fast developments in artificial breeding and aquaculture, new varieties of razor clams for artificial breeding are still severely lacking. At present, limited research has reported on growth-related genes in *S. constricta*, including *IGFBP* (Xie et al., 2015), *MSTN* (Niu et al., 2015), and *GRB2* (Zhao et al., 2018). There is thus a need to study growth-related genes and carry out molecular breeding of high-yield new varieties to support the sustainable development of the clam aquaculture industry. In this study, we identified the promotor and exon of *S. constricta BMP*7 (*Sc-BMP*7) gene, and detected its expression profiles in different tissues and developmental stages. Furthermore, we also analyzed the association of *Sc-BMP*7 SNPs with growth traits, thus providing the basis for screening candidate genes for growth traits and for studying the molecular mechanisms of growth regulation.

## Materials and Methods

### Experimental Animals and Sample Collection

Adult clams (shell length 50 ± 5 mm, total weight 7.0 ± 1.0 g) were obtained from Yinzhou Danyan Aquaculture Field in Ningbo, China, for cloning and gene expression analysis of *Sc-BMP7*. Eleven tissues, including mantle (pallial zone, marginal zone and central zone), adductor muscle, digestive gland, foot, gill, blood, gonad, and siphon were dissected, frozen immediately in liquid nitrogen, and then stored at −80°C. Embryos/larvae were cultured in 13‰ salinity seawater, fed with golden-brown algae, and collected at 10 developmental stages (unfertilized mature egg, fertilized egg, 4-cell embryo, blastula, gastrula, trochophore, D-shaped larva, umbo larva, eyespot larva, juvenile clam) and preserved at −80°C.

A total of 246 adult clams were collected to screen for *Sc-BMP*7 SNPs. 122 individuals from Yongle NO 1 strain (fast-growing strain, selected for four generations by our team from Changle population, Fujian Province, China) and 124 individuals from Lianjiang population (wild population from Lianjiang county, Fujian Province, China) were randomly sampled. The two groups were cultured in the same growing environmental conditions, and the main growth traits (shell length, shell width, shell height, and total weight) were measured. The foot and mantle were dissected, frozen immediately in liquid nitrogen, and then stored at −80°C.

### Cloning of Full-Length cDNA and Promoter

Total RNA was extracted from the mantle using Trizol reagent (Sangon, China). RNA integrity was determined by formaldehyde-denatured 1.2% agarose gel electrophoresis and staining, and the quality and quantity were assessed by ultraviolet spectrophotometry. First-strand cDNA was synthesized using SMART RACE reagent (Clontech, United States).

Expressed sequence tag (EST) sequences of *Sc-BMP*7 gene were retrieved from the razor clam transcriptome in the SRA database (NCBI) with accession number SRP2162898. Primers for 5’-RACE (Sc-BMP7-F1) and 3’-RACE (Sc-BMP7-R1) were designed (Table 1). Polymerase chain reaction (PCR) products were purified using a gel extraction kit (Tiangen, China) and then cloned into the T1 vector (TaKaRa, Japan). The vector was transformed into T1 cells (Tiangen) according to the manufacturer’s protocols, and positive clones were sequenced.

**TABLE 1:** Primers and sequences of the experiments.

Primers	Sequences (5′-3′)	Applications
Sc-BMP7-F1	GAATACCATCGGAAGTCCTCGGTCAGTC	3′-RACE
Sc-BMP7-R1	CCATCTGGGTGAATGAACTTGTCGTCGG	5′-RACE
Sc-BMP7-F2	ATACGCAAAACCAATATGGAGGC	Verifying the sequence of cDNA
Sc-BMP7-R2	AGAGGCAGTAATAACACAAGACAGG	Verifying the sequence of cDNA
Sc-BMP7-F3	CCAACTGACAGACAACAGGTAGAA	Cloning of intron
Sc-BMP7-R3	AGATGTTAGCGTCCTGGATTGC	Cloning of intron
Sc-BMP7-F4	CACAGGACAAGACATTGGAACC	Cloning of intron
Sc-BMP7-R4	CCACAGAAGAACGCAGGATAAC	Cloning of intron
Sc-BMP7-F5	AGATGCTACATTCGTTGGTGAGA	Cloning of intron
Sc-BMP7-R5	CGAAATACAATACTTGGATGGACG	Cloning of intron
Sc-BMP7-R6	TATTGTTCACGGGTCGGG	Cloning of promoter
Sc-BMP7-R7	TTGGTCTGTTTGTCCTAATGGC	Cloning of promoter
RBF	TGTGCGTGGATTTCCTTTG	qRT-PCR
RBR	TGAGTCGGATTTCTGGTTCG	qRT-PCR
18S-F	TCGGTTCTATTGCGTTGGTTTT	qRT-PCR
18S-R	CAGTTGGCATCG TTTATGGTCA	qRT-PCR
SBMP7-F1	CGAACCAGAAATCCGACTC	SNP
SBMP7-R1	GTGCGTAAGTGCGTAAGACC	SNP
SBMP7-F2	GCATTCCTGTTAGCCATTTAGTTG	SNP
SBMP7-R2	TGAGTCGGATTTCTGGTTCG	SNP
BF	GCGTAATACGACTCACTATAGG GCTTCTACTACTGGGGTGGTG	RNAi
BR	GCGTAATACGACTCACTATAG GGCGGTAGTGACGCAACAATT	RNAi
HBF	GCGTAATACGACTCACTATA GGGACACGACTTGACACGGTAT	RNAi
HBR	GCGTAATACGACTCACTATA GGGGCGACAGTTTCTGGGTAGT	RNAi

To confirm the accuracy of the cloning and sequencing, the full-length cDNA was reamplified using a pair of specific primers, Sc-BMP7-F2 and Sc-BMP7-R2 (Table 1), designed based on the *Sc-BMP*7 cDNA. The PCR products were cloned and sequenced following the procedures described above.

We designed the reverse primer using Primer 5 software based on the *Sc-BMP*7 cDNA. The PCR products were cloned and sequenced following the procedures described above, using a genome walker kit (TaKaRa). The possible core promoter region and potential transcription factor binding sites were predicted by the online software BDGP^1^ and Alibaba2^2^. The CpG island was predicted using the online analysis software Meth primer^3^.

### Sequence and Phylogenetic Analysis

Sequences were spliced using the National Center for Biotechnology Information database BLAST algorithm^4^. The deduced amino acid sequence was analyzed using the simple modular architecture research tool (SMART)^5^ to predict conserved domains. The presence and location of the signal peptide and cleavage sites in the amino acid sequence were predicted by SignalP 4.0 server^6^. Multiple alignments of BMP7 proteins between *S. constricta* and other species were performed using the ClustalW2 multiple alignment program^7^. A phylogenetic tree was constructed by the neighbor-joining method with MEGA 6.0.

### Quantitative Analysis

The expression profiles of *Sc-BMP*7 during different developmental stages (n > 500, three sets of samples per stage) and in different adult tissues (*n* = 4, four sets of samples per tissue) were analyzed using real time quantitative reverse transcription PCR (qRT-PCR). Total RNA was extracted from the samples as described above. Primers RBF and RBR (Table 1) were designed using Primer 5 and 18S rRNA was used as an internal reference. PCR was conducted using a 7500 Fast Real-Time PCR machine (ABI, United States). The relative value of 2^–ΔΔ*Ct*^ was adopted for data processing. Quantitative differences in fluorescence results were analyzed by SPSS 20.0. One way ANOVA was adopted to compare the difference of these groups. A p-value less than 0.05 (*P* < 0.05) was considered as statistical significance.

### Shell Repair Experiment

48 clams (shell length 40 ± 5 mm) were divided randomly into three treatment groups (*n* = 8 per group) and three control groups (*n* = 8 per group). Shell incision was carried out after holding for 3 days. In the treatment groups, a V-shape cut was performed on both sides of the clam’s shells using scissors, but the mantle was not damaged during the operation. Clams in the control groups remained untreated. One treatment group and one control group were then cultured together in the same tank used sterilized seawater, with a total of three tanks. All clams were fed with the microalgae *Isochrysis galbana* in the morning and evening. Three shells were then sampled from the treatment group and control group at 2, 4, 8, 12, 24, 48, and 96 h, and at 7 days, respectively. The outer mantle tissue was dissected at the V-shaped notch and total RNA was extracted for qRT-PCR, as described above.

### Effect of Ca^2+^ on BMP7 Gene Expression

A total of 60 clams (shell length 40 ± 5 mm) were divided randomly into six groups. The control group was NSG (normal seawater group). CFG (calcium free group), Ca^2+^ levels was lower than normal levels (seawater plus EDTA-Na_2_ to a final concentration of 250 mg/L), and LCG (low calcium free group), MCG (middle calcium group), HCG (high calcium group), and VHCG (very high calcium free group) groups, were levels higher than normal seawater (CaCl_2_ added to final concentrations of 100, 200, 300, and 400 mg/L, respectively). The clams were cultured in the same water environment (salinity 25‰, pH 8.0 ± 0.3 and temperature 25°C) for 3 weeks in 50 L barrels and fed *I. galbana* in the morning and evening. The seawater was changed every day. The mantle and gill tissues were then dissected, and total RNA was extracted for qRT-PCR analysis, as described above.

### RNA Interference (RNAi) and Enzyme-Linked Immunosorbent Assay (ELISA)

RNAi was performed to examine the role of *Sc-BMP7* in shell growth. The specific primers (Table 1) were designed based on the *Sc-BMP*7 cDNA and used to amplify the specific sequences. The *T. granosa* Hb gene (which is not expressed in *S. constricta*), and DEPC H_2_O was used as a control. Ten individuals were used in each treatment. RNAi was conducted according to Yan et al. (2014). *Sc-BMP*7 double-stranded (ds)RNA was diluted to 1 μg/μL with DEPC H_2_O, and 80 μL was injected into the adductor muscle of *S. constricta* (1-year-old, shell length 48 ± 5 mm), followed by the same dose 3 days after the first injection. Clams in the control groups were injected with the same volume of DEPC H_2_O or 1 μg/μL of hemoglobin-dsRNA (Hb-dsRNA) in DEPC H_2_O. Total RNA was extracted from the mantle pallium and edge 3 days after the third injection. The expression levels of the *Sc-BMP*7 gene after RNAi were measured by qRT-PCR using *18sRNA* as the internal reference.

Sc-BMP7 proteins of ten samples from each treatment group above were detected by ELISA using the shellfish BMP-7 ELISA kit (Jianglai, China). Statistical analysis of results between RNAi group and control group were used SPSS 20.0. ANOVA was adopted to compare the difference of these groups. A *p*-value less than 0.05 (*P* < 0.05) was considered as statistical significance.

### Exon SNPs

The *Sc-BMP*7 cDNA was used to design the primers SBMP7-F1, SBMP7-R1, SBMP7-F2, and SBMP7-R2 (Table 1). Total RNA was extracted from the samples as described above. The PCR products were verified by direct sequencing and sequence alignment was conducted using MEGA6.0 software. The relationships between SNPs and growth traits were analyzed using SPSS20.0. ANOVA was adopted to compare the difference of these genotypes. Linkage disequilibrium (LD) and haplotype were analyzed using SHEsis analysis software^8^.

## Results

### Sequence Analysis of Sc-BMP7 Gene

**FIGURE 1:**
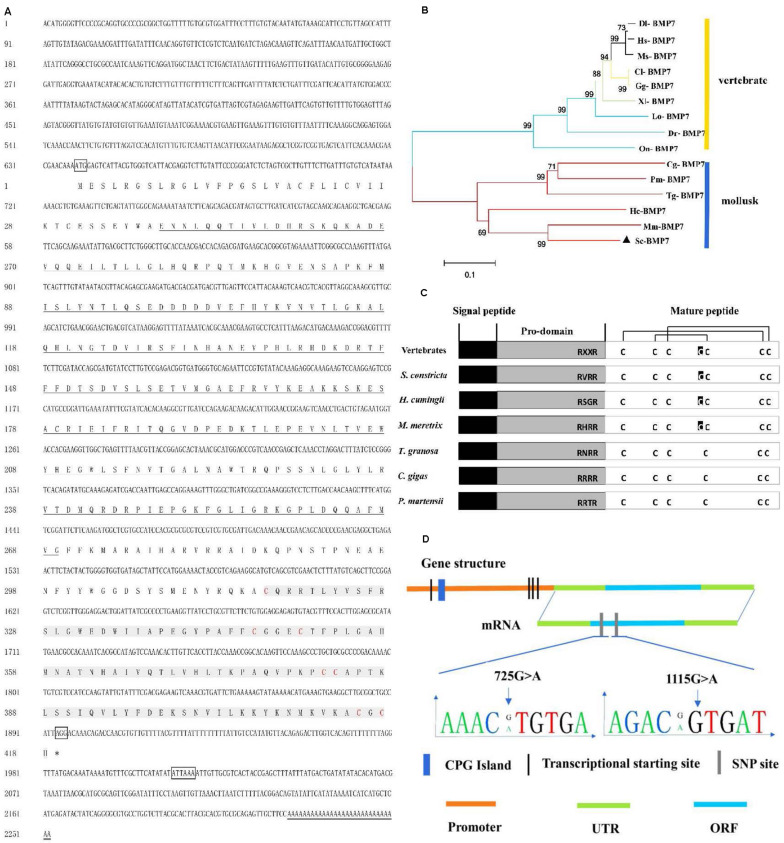
Analysis of sequence and structure of *Sc-BMP*7 gene. **(A)** The full-length cDNA and deduced amino acid sequence of Sc-BMP7. The letters boxes are the start codon, the stop codon and the polyadenylation signal sequence, the ^∗^ represents the end of the protein translation, the double line is the poly A. the bold underlined parts are the propeptide, shaded gray part is the signal peptide, red letters are cysteine residues. **(B)** Neighbor-joining phylogenetic tree of BMP7 between *S. constricta* and other species using MEGA6.0 software. The abbreviation of BMP7 and the GenBank accession numbers used to construct phylogenetic tree are given in Table 2. **(C)** Structure of Sc-BMP7 and selected BMP7 genes from mollusks and vertebrates. **(D)** Gene structure of Sc-BMP7 gene.

The deduced Sc-BMP7 protein contained 418 amino acid residues encoded by 2,247 nucleotides. The cDNA also contained a 5’ untranslated region (UTR) of 638 nucleotides and a 3’ UTR of 117 nucleotides (Figure 1A). The calculated molecular mass of the deduced mature Sc-BMP7 was 47.70 kDa, and the theoretical isoelectric point was 7.59. Amino acid sequence analysis showed that Sc-BMP7 contained a signal peptide (1–32 aa), a prodomain (33–283 aa), and a mature peptide (284–418 aa). The mature protein, which was produced by cleaving off the prodomain in the putative maturation site Arg-X-X-Arg, consisted of 135 amino acids, and a TGF-β family domain (318–429 aa) with seven conserved cysteine residues (Figure 1C). PCR amplification was carried out using two pairs of primers. After assembly, a proximal promoter of 2,252 bp was obtained using the genomic walking method. The promoter contained a CpG island, 203 transcription factor binding sites, and four potential transcription initiation sites.

Multiple comparisons with the BMP7 of mollusks and model animals showed that Sc-BMP7 shared the highest identity (68%) with BMP7 of *Meretrix meretrix*, and 39%–55% similarity with others. The abbreviations of BMP7 and the GenBank accession numbers used to construct the phylogenetic tree are shown in Table 2. The phylogenetic tree, constructed using the neighbor-joining method, showed that BMP7 protein could be divided into two groups (Figure 1B), one containing all shellfish, and another comprising mammals, reptiles, and fish. *M. meretrix* BMP7 was firstly clustered with Sc-BMP7 in the former group.

**TABLE 2:** Species and GenBank accession numbers of BMP7s sequence used for multiple alignment and phylogenetic analysis.

Species	Abbreviation type	GenBank no.	Size	Homology (%)
*S. constricta*	Sc-BMP7	MH822127	418	-
*Meretrix meretrix*	Mm-BMP7	ALG64478.1	418	68
*Hyriopsis cumingii*	Hc-BMP7	AJI77173.1	428	55
*Tegillarca granosa*	Tg- BMP7	AFP57673.1	425	47
*Pinctada martensii*	Pm-BMP7	AGS32053.1	431	49
*Crassostrea gigas*	Cg-BMP7	EKC34211.1	406	47
*Lepisosteus oculatus*	Lo-BMP7	XP_006639569.1	425	41
*Oreochromis niloticus*	On-BMP7	XP_003439028.1	427	42
*Danio rerio*	Dr-BMP7	AAF17558.1	432	39
*Xenopus laevis*	Xl-BMP7	AAI08478.1	424	39
*Gallus gallus*	Gg-BMP7	XP_417496.5	465	41
*Columba livia*	Cl-BMP7	KK25127.1	346	47
*Mus musculus*	Ms-BMP7	NP_031583.2	430	42
*Homo sapiens*	Hs-BMP7	NP_001710.1	431	42
*Delphinapterus leucas*	Dl-BMP7	XP_022453203.1	431	42

### Quantitative Expression Analysis of Sc-BMP7

**FIGURE 2:**
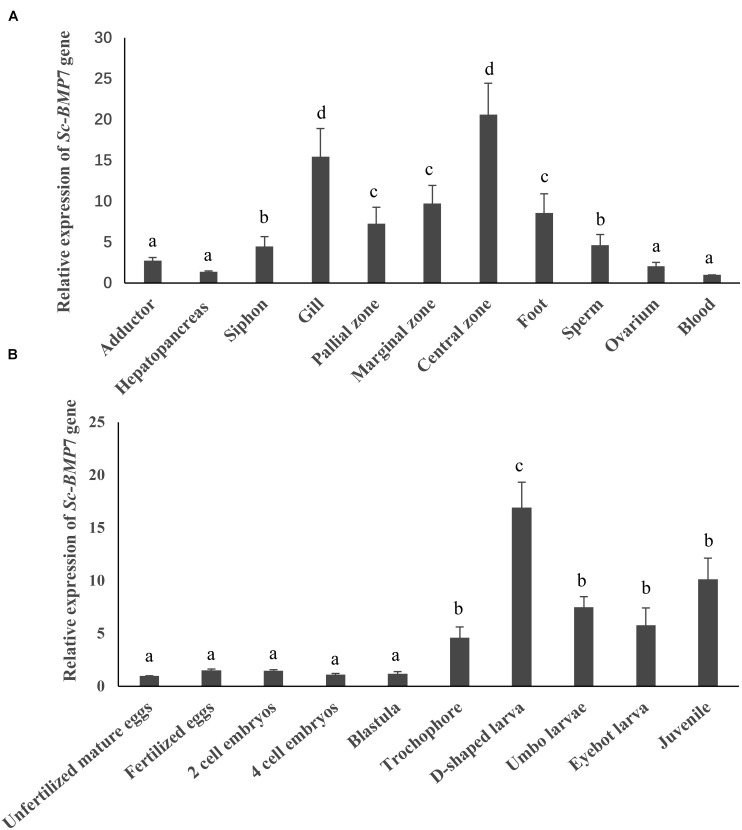
Quantitative expression analysis of *Sc-BMP*7, **(A)** Gene expression difference analysis of *Sc-BMP*7 in different tissues. The same letters indicate no difference in the level of expression, whereas different letters indicate significant differences in expression levels among various tissues (*P* < 0.05, based on ANOVA, *n* = 4). **(B)** Gene expression difference analysis of *Sc-BMP*7 in different developmental stages. The same letters indicate no difference in the level of expression, whereas different letters indicate significant differences in expression levels among various developmental stages (*P* < 0.05, based on ANOVA, *n* > 500).

Tissue and developmental stage-specific expression of *Sc-BMP7* were determined by qRT-PCR. *Sc-BMP7* gene expression levels were high in the gills and mantle (*P* < 0.05), especially in the central zone of mantle (Figure 2A). In terms of developmental stage, *Sc-BMP7* expression levels were very low before the trochophore stage, including unfertilized mature eggs, fertilized eggs, 4-cell embryos, blastulae, and gastrulae, but gradually increased in the subsequent developmental stages, with the highest levels in D-shaped larvae (*P* < 0.01) (Figure 2B).

### Shell Repair Experiment

**FIGURE 3:**
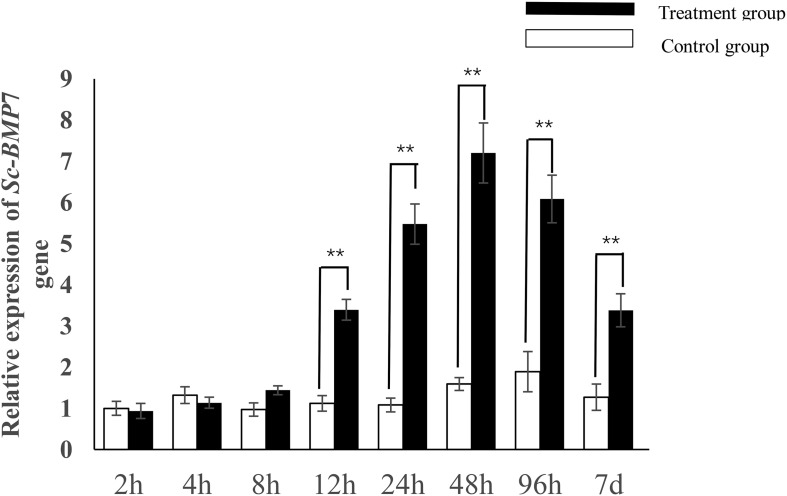
Quantitative expression analysis of *Sc-BMP*7 after shell notching. ^∗∗^ represents extremely significant differences, *P* < 0.01.

*Sc-BMP7* expression showed no significant increase to 8 h after incision but increased significantly after 12 h (*P* < 0.05) and peaked after 48 h (Figure 3). *Sc-BMP7* expression then began to decline until the seventh day, with extremely significant difference between two groups (*P* < 0.01).

### Effect of Ca^2+^ on BMP7 Gene Expression

**FIGURE 4:**
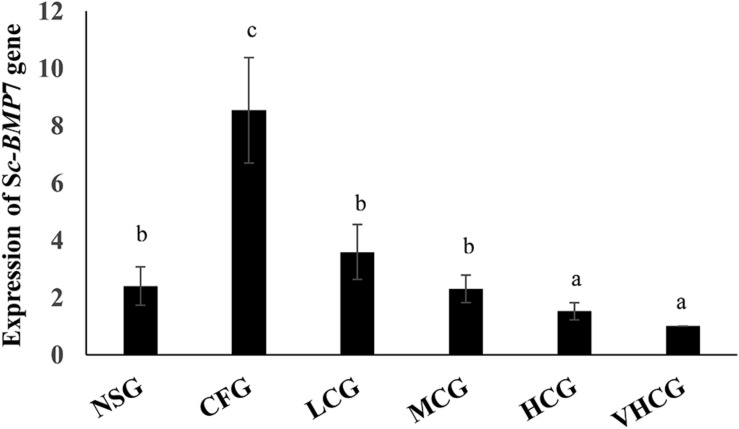
Quantitative expression analysis of *Sc-BMP*7 in seawater with different Ca^2+^ concentration. Different letters represent significant differences, *P* < 0.05. NSG: normal seawater group; CFG: calcium free group, add EDTA-Na_2_ to a final concentration of 250 mg/L; LCG, MCG, HCG, and VHCG represent CaCl_2_ added to final Ca^2+^ concentrations of 100, 200, 300, and 400 mg/L, respectively.

*Sc-BMP*7 expression in the mantle was significantly higher in the CFG group compared with the NSG group. *Sc-BMP*7 expression in the mantle was increased by the addition of 100 mg/L CaCl_2_, and was unaffected by the addition of 200 mg/L or 300 mg/L CaCl_2_ to the seawater. However, the expression levels were reduced after the addition of 400 mg/L CaCl_2_ compared with normal seawater (Figure 4).

### Role of Sc-BMP7 in Shell Growth

**FIGURE 5:**
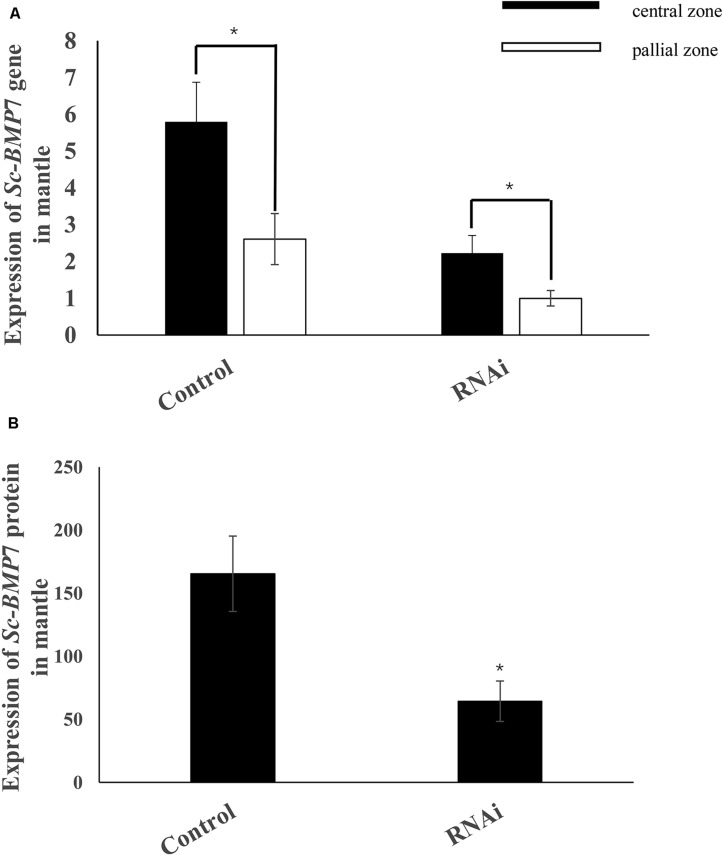
Expression analysis of *Sc-BMP*7. **(A)** Expression analysis of *Sc-BMP*7 gene in the central zone and pallial zone of mantle; **(B)** Expression analysis of *Sc-BMP*7 protein in mantle. ^∗^ represents significant differences (*P* < 0.05, based on ANOVA).

We further investigated the function of *Sc-BMP*7 in shell biomineralization *in vivo* using RNAi to inhibit the expression of *Sc-BMP*7 gene. We measured *Sc-BMP*7 mRNA levels in the mantle pallium and the mantle edge using qRT-PCR. *Sc-BMP*7 gene expression levels in the RNAi group were downregulated to approximately 38% in the mantle pallium and 29% in the mantle edge compared with control levels (Figure 5A). Sc-BMP7 protein levels were significantly lower in the *Sc-BMP*7 RNAi group (64.31 pg/mL) compared with the control group (165.37 pg/mL) (Figure 5B).

### Growth-Related SNPs in Sc-BMP7 Exon

Sequence comparisons detected 20 SNPs in *Sc-BMP*7. All the SNPs were synonymous mutations, with A/G transversions accounting for 50%. Of these, nine SNPs were in the 5’ UTR or 3’ UTR, and others in the ORF. The associations between its SNPs and growth traits are shown in Table 3. Five SNPs (413G > A, 725G > A, 986G > A, 1017A > C, 1115G > A) were associated with growth traits in the Yongle NO1 strain. The genotype, allele frequencies and polymorphism information content (PIC) values in Yongle NO1 strain are shown in Table 4. Furthermore, analysis of polymorphic parameters indicated that all these SNPs were moderately polymorphic (0.25 < PIC < 0.5).

**TABLE 3:** Analysis of association between SNPs and growth traits in two groups.

Group	SNP sites	Allele and frequency		PIC	Ne	Ho	He	HWEp
“Yongle NO1” strain	413 G > A	G 0.6148	A 0.3852	0.3614	1.8999	0.7049	0.5244	0.0001
	725 G > A	G 0.6680	A 0.3320	0.3451	1.7970	0.5656	0.5546	0.7857
	986 G > A	G 0.9262	A 0.0738	0.1273	1.1583	0.9016	0.8628	0.0001
	1017 A > C	A 0.8607	C 0.1393	0.2110	1.3155	0.8525	0.7592	0.0002
	1115 G > A	G 0.8770	A 0.1230	0.1924	1.2750	0.8852	0.7834	0.0001
“Lianjiang” population	725 G > A	G 0.6492	A 0.3508	0.3517	1.8365	0.6371	0.5427	0.0218
	1115 G > A	G 0.7903	A 0.2097	0.2765	1.4957	0.8065	0.6672	0.0001

**TABLE 4:** The polymorphic parameters of SNP sites in *Sc-BMP*7 gene.

Group	Site	Genotype	N	Frequencies (%)	Shell length (mm)	Shell width (mm)	Shell height (mm)	Total weight (g)
“Yongle NO1” strain	413 G > A	AA	29	23.77	50.24 ± 3.25	11.68 ± 1.24 ab	17.04 ± 1.16	6.62 ± 1.39 ab
		AG	36	29.50	50.25 ± 4.40	12.01 ± 1.53 b	17.29 ± 1.52	6.99 ± 1.91 b
		GG	57	46.73	48.63 ± 4.42	11.35 ± 1.22 a	16.50 ± 2.51	6.13 ± 1.59 a
	725 G > A	AA	14	11.48	47.73 ± 3.80	10.89 ± 1.05 a	15.35 ± 4.26 a	5.68 ± 1.42 a
		GA	53	43.44	49.37 ± 4.13	11.46 ± 1.18 ab	17.05 ± 1.40 b	6.44 ± 1.54 b
		GG	55	45.08	50.05 ± 4.32	11.95 ± 1.47 b	17.07 ± 1.44 b	6.78 ± 1.82 b
	986 G > A	AA	3	2.46	46.89 ± 5.96	10.24 ± 1.21 a	15.96 ± 1.69	4.97 ± 1.88 a
		GA	107	87.70	50.39 ± 3.47	11.72 ± 1.21 b	17.43 ± 1.22	6.83 ± 1.52 b
		GG	12	9.84	49.50 ± 4.14	11.63 ± 1.33 b	16.82 ± 2.70	6.51 ± 1.64 b
	1017 A > C	AA	96	78.70	48.97 ± 4.18 a	11.60 ± 1.38	16.63 ± 2.11	6.37 ± 1.67
		AC	18	14.75	52.40 ± 2.47 b	11.99 ± 0.98	17.95 ± 0.83	7.37 ± 1.05 b
		CC	8	6.55	49.83 ± 4.96 b	11.08 ± 1.29	17.05 ± 1.78	6.20 ± 1.91
	1115 G > A	AA	8	6.66	48.93 ± 3.24 a	11.47 ± 1.24	16.74 ± 1.39	6.26 ± 1.30 a
		GA	14	11.48	52.45 ± 2.94 b	12.11 ± 0.91	17.98 ± 0.89	7.67 ± 1.06 b
		GG	100	81.96	49.17 ± 4.18 a	11.57 ± 1.38	16.71 ± 2.12	6.37 ± 1.68 a
“Lianjiang” population	725 G > A	AA	21	16.93	30.62 ± 5.05	6.79 ± 1.39 a	10.33 ± 1.84 a	1.53 ± 0.84 a
		AG	45	36.29	32.81 ± 4.87	7.54 ± 1.24 b	11.06 ± 1.55 b	1.91 ± 0.77 b
		GG	58	56.78	32.67 ± 3.85	7.45 ± 1.05 b	10.99 ± 1.25 ab	1.82 ± 0.60 ab
	1115 G > A	AA	14	11.29	31.03 ± 4.14 a	6.94 ± 0.98 a	10.52 ± 1.48 a	1.57 ± 0.57 a
		AG	24	19.35	34.64 ± 3.35 b	7.92 ± 1.054 b	11.60 ± 1.20 b	2.19 ± 0.75 b
		GG	86	69.36	31.90 ± 4.51 a	7.28 ± 1.24 ab	10.76 ± 1.51 a	1.72 ± 0.69 a

*Different letters represent significant differences, *P* < 0.05.*

SNPs 725G > A and 1115G > A were significantly associated with growth traits in Lianjiang population (Figure 1D). Shell width, shell height, and total weight were significantly higher in clams with the GA compared with the AA genotype of 725G > A in the two groups. Shell length and total weight were significantly higher in clams with heterozygous GA type 1115G > A, compared with homozygous AA or GG in both groups (*P* < 0.05; Table 3). Meanwhile, the frequency of the dominant AG genotype of 725G > A was significantly higher in Yongle NO1 strain than in Lianjiang population, while the AA genotype was less common in Yongle NO1 strain compared with Lianjiang population, possibly related to the breeding of Yongle NO 1 strain.

LD analysis using SHEsis online software showed linkage in 725G > A and 1115G > A in both strains (Table 5). LD and haplotypes across SNPs are shown in Table 5. There were two haplotypes of the *BMP*7 gene. The frequencies of the AG and GA haplotypes in Yongle NO1 strain were significantly higher than in Lianjiang population, but there was no significant difference between the two groups in terms of the AA and GG haplotypes.

**TABLE 5:** Haplotype analysis of two SNP sites in *Sc-BMP*7 gene.

Haplotype	Sequence	“Yongle NO1” strain (frequency)	“Lianjiang” population (frequency)	χ^2^ (*P* value)
a	AA	22.76 (0.092)	15.18 (0.062)	1.51 (0.21)
b	AG	29.24 (0.118)	65.82 (0.270)	18.19 (2.02e-05)
c	GG	131.76 (0.259)	148.18 (0.607)	2.89 (0.09)
d	GA	64.24 (0.259)	14.82 (0.061)	35.863 (2.18e-09)

## Discussion

The results of our current study showed that the *Sc-BMP*7 gene was highly similar in sequence size to *BMP*7 genes from some species (Dong, 2012; Xu et al., 2018), but differed from those of *P. martensii* (Yan et al., 2014) and *M. meretrix*. The full-length cDNA may have differed from the two homologous genes because of the existence of the additional homologous gene, *BMP7b* (Shawi and Serluca, 2008; Fan et al., 2018). Previous research showed that all BMPs were synthesized as large precursors (Xiao et al., 2007; Nelsen and Christian, 2009). Mature BMPs in vertebrates share seven conserved cysteines, which can build a cystine knot, active hetero- or homodimers by forming intrachain disulfide bonds or interchain disulfide bonds (Fairlie et al., 2001; Wozney, 2002). This phenomenon was also found in the Sc-BMP7 protein, but only six conserved cysteines were found in *C. gigas* and *T. granosa* (Dong, 2012). This may indicate that Sc-BMP7 may possess certain different functions in mollusks.

*BMP*7 plays an important role in early embryonic development, organ formation, and development (Cook and Rueger, 1996; Bragdon et al., 2011). Bivalves have similar shell-formation processes, and shells occur at an early stage of embryonic development. Development of the shell can be divided into five stages (Kin et al., 2009). The shell begins to form prodissoconch I during the larval stage, followed by prodissoconch II (Miyazaki et al., 2010) in D-larvae. The prodissoconch I is completely formed during gastrula formation, and the prodissoconch II begins to form with formation of the velum (Kakoi et al., 2008). In this study, *Sc-BMP*7 expression was lower before the trochophore stage, probably because cell division is the main event in early embryonic development. However, *BMP7* expression began to increase in D-shaped larva, with the beginning of shell formation (*P* < 0.05), like the situation in *T. granosa* (Dong, 2012). Higher expression of *Sc-BMP7* in the juvenile stage then reflects the fast growth of razor clams, and the rapid development of organs and shells at this stage (Wang and Wang, 2008).

Previous studies on mollusks found higher expression levels of *BMP*7 in the gills and mantle (Dong, 2012; Lin, 2014; Yan et al., 2014; Fan et al., 2018). In this study, *Sc-BMP*7 was expressed in all tissues, especially showed higher expression in the gills and mantle. Gills are the respiratory and filter-feeding organs in bivalves and consume large amounts of energy during the processes of food filtration and gas exchange (Gajaraville et al., 1990; Cui et al., 2006), associated with metabolic vigor and rapid cell proliferation, potentially requiring more BMP7 to regulate cell function. The mantle is the main organ for shell formation and regulates the extracellular growth of crystals, and secretes matrix proteins (Awaji and Machii, 2011; Liu and Li, 2013). The mantle can be divided into marginal, pallial, and central zones according to its different functions (Daisuke et al., 2014; Fan et al., 2018). The *Sc-BMP*7 gene expression is higher in the central zone than in the other zones. Formation of the prismatic layer of the shell mainly depends on the marginal zone of the mantle, while the central zone secretes the pearl layer because the calcium ion channels (Barry and Diamond, 1971; Shi et al., 2012). *Sc-BMP*7 may play a crucial role in nacre formation of the shell, as well as being involved in formation of the prismatic layer. The results of the gene expression by calcium concentration showed that the *Sc-BMP*7 expression in low calcium environment was increased compared with normal seawater. Probably because biomineralization requires a certain amount of Ca^2+^. Ca^2+^ was absorbed mainly through the digestive organs and gills, and then transported to the mantle to participate in shell formation (Luo et al., 2010). *Sc-BMP*7 gene expression increased to maintain mineralization with a small amount of calcium ion when the Ca^2+^ concentration in seawater decreased. In addition, mollusks have a stable calcium metabolism system, the secretion of calcium regulated by matrix protein, as an essential factor of shell formation. Excessive calcium ions may affect normal shell growth. When the concentration of Ca^2+^ in seawater increases substantially, *Sc-BMP*7 gene was low expressed to stabilize the biomineralization in razor clam. The result is similar to the study of *BMP*3 in *P. martensii* (Zhou, 2016).

We further elucidated the role of *Sc-BMP*7 in shell formation in razor clams by RNAi and ELISA analysis. *Sc-BMP*7 expression decreased significantly following RNAi, by approximately 62% in the pallial zone and 71% in the central zone of the mantle. Meanwhile, Sc-BMP7 protein also decreased significantly by approximately 52%. BMPs are not only a bone-inducing factor, and it is also a major component of bone (Urist, 1965; Wozney, 2002). In mollusks, CaCO_3_ polymorph, and size and shape of the crystals are controlled by matrix proteins secreted by mantle epidermal cells (Marin and Luquet, 2004; Liu and Li, 2013). In addition, preosteoblast differentiation can be induced by nacre, especially the water soluble matrix fraction of nacre, finally leading to bone formation (Silve et al., 1992; Mouriès et al., 2002), and it is also involved in the activation of mantle cells in mollusks (Sud et al., 2001). The *BMP*7 gene has been shown to be highly expressed in the mantle in various shellfish. Furthermore, inhibition of the *BMP*7 gene by RNAi disrupted the growth of aragonite tablets and resulted in holes in the calcite crystals in the mantle of *P. martensii*, indicating that *BMP7* participated in the formation of nacre and the prismatic layer (Yan et al., 2014). In the current study, RNAi treatment decreased the gene and protein expression of *Sc-BMP7*, potentially resulting in decreased activation and secretion of matrix proteins by the mantle epidermal cells.

Molecular markers can be used to allow selection at the molecular level during assisted breeding, thus greatly improving the breeding efficiency. SNP is the most widely distributed molecular marker in genome and has been used in molecular breeding. *BMP*7 has previously been identified as a candidate growth-related gene in SNP studies in cattle (Wang, 2009) and chickens (Chen et al., 2013; Wang et al., 2018), but not in mollusks. The current study detected 20 SNPs in the *Sc-BMP*7 cDNA sequence in Yongle NO1 strain and Lianjiang population, implying a high frequency of SNPs. All the SNPs showed moderate (0.25 < PIC < 0.5) or low polymorphism (PIC < 0.25), presumably because SNP markers are typically biallelic making it difficult to show high polymorphism, as seen for simple sequence repeat (SSR) markers (Hubert et al., 2009).

Analysis of the SNPs identified two (725A > G and 1115A > G) that were associated with growth traits in both groups. GA at 725A > G was significantly associated with shell height and total weight compared with AA, while GA at 1115G > A was significantly associated with shell length and total weight compared with GG and AA in both Yongle NO1 and Lianjiang groups. Furthermore, the two SNPs were located within the coding region of the *Sc-BMP*7 gene with no amino acid changes. Various studies have shown that synonymous mutations can regulate gene transcription and translation by affecting transcriptional efficiency or changing the mRNA molecules and spatial structure of the protein (Greenwood and Kelsoe, 2003; Capon et al., 2004; Kimchi-Sarfaty et al., 2007).

In conclusion, we got the cDNA and promoter of *Sc-BMP*7 gene and then analyzed the sequence characteristics and phylogenetic relationship. Analysis of tissue- and development-specific expression demonstrated *Sc-BMP*7 mRNA was the highest in the central zone of mantle (*P* < 0.05) and D-shaped larva (*P* < 0.05), suggesting that it may be involved in the formation and growth of shells. Further results of shell repair experiment and RNAi indicated that *Sc-BMP*7 gene plays a vital role in repairing shell damage and its function is affected by the Ca^2+^ concentration in seawater. Moreover, association analysis identified two shared SNPs in exon of *Sc-BMP*7 gene from 246 individuals of two groups. These results of the present study would contribute to clarify the role of *Sc-BMP*7 in the regulation of growth and shell formation, and provide growth-related markers for molecular marker assisted breeding in *S. constricta*.

## Data Availability Statement

The BMP7 SNP data has been deposited into the European Variation Archive (EVA) with the project accession PRJEB39579 and analysis accession ERZ1468016.

## Author Contributions

YD and ZL conceived and designed the project. HY collected the samples and contributed reagents. JZ and BC performed the experiments and data analysis. YD and JZ wrote and revised the manuscript. All authors read and approved the final manuscript.

## Conflict of Interest

The authors declare that the research was conducted in the absence of any commercial or financial relationships that could be construed as a potential conflict of interest.

## Footnotes

1 http://www.fruitfly.org/seq_tools/promoter.html

2 http://www.gene-regulation.com/pub/programs/alibaba2/index.html

3 http://www.urogene.org/methprimer/index1.html

4 http://www.ncbi.nlm.nih.gov/blast/

5 http://smart.embl-heidelberg.de

6 http://www.cbs.dtu. dk/services/SignalP/

7 http://www.ebi.ac.uk/Tools/msa/clustalw2/

8 http://analysis.bio-x.cn/myAnalysis.php
